# Supplementary material for: Metabolic potential of uncultured bacteria and archaea associated with petroleum seepage in deep-sea sediments
Source: Nat Commun. 2019 Apr 18;10:1816. doi: 10.1038/s41467-019-09747-0 (PMC6472368; doi:10.1038/s41467-019-09747-0)
Supplement: Supplementary file 1 — Supplementary Information [file 41467_2019_9747_MOESM1_ESM.pdf]

Supplementary materials

Metabolic potential of uncultured bacteria and archaea associated with petroleum seepage in deep-sea sediments

Xiyang Dong [1,*], Chris Greening [2], Jayne E. Rattray [1], Anirban Chakraborty [1], Maria Chuvochina [2], Daisuke Mayumi [1,3], Jan Dolfing [4], Carmen Li [1], James M. Brooks [5], Bernie B. Bernard [5], Ryan A. Groves[1], Ian A. Lewis [1], Casey R.J. Hubert [1,*]

[1] Department of Biological Sciences, University of Calgary, Calgary, T2N 1N4, Canada

[2] School of Biological Sciences, Monash University, Clayton, VIC 3800, Australia

[3] Institute for Geo-Resources and Environment, Geological Survey of Japan, National Institute of Advanced Industrial Science and Technology (AIST), 1-1-1 Higashi, Tsukuba, 305-8567, Japan

[4] School of Engineering, Newcastle University, Newcastle upon Tyne, NE1 7RU, United Kingdom

[5] TDI Brooks International, College Station, Texas, TX 77845, USA

* Corresponding authors. E-mail: xiyang.dong@ucalgary.ca (X. Dong), chubert@ucalgary.ca (C. R.J. Hubert).

List of contents

Supplementary Note 1

Supplementary Note 2

Supplementary Table 1 Sample metadata and shotgun sequencing results.

Supplementary Table 2 Alpha diversity estimates based on 16S rRNA gene amplicon sequencing.

Supplementary Table 3 Functional capacity based on normalized raw read count of genes (counts per million reads, CPM) encoding key metabolic enzymes.

Supplementary Figure 1 Map of the Eastern Gulf of Mexico (GoM) showing the studied three sampling locations (E26, E29 and E44) and bathymetry of the study area.

Supplementary Figure 2 Phylum-level community composition of classified bacterial and archaeal16S rRNA gene reads from separate bacterial and archaeal amplicon libraries. Left panel: bacterial composition; right panel: archaeal composition. Detailed numbers can be found in Supplementary Data 1.

Supplementary Figure 3 Phylogenetic relationship of putative glycyl-radical enzymes in MAGs with those of akyl-/arylalkylsuccinate synthases. Reference sequences show canonical alkane succinate synthase (AssA), 4-hydroxylbenzyl succinate synthase (HbsA), benzyl succinate synthase (BssA), and homologous putative alkane-degrading enzymes from *Vallitalea guaymasensis* L81 and *Archaeoglobus fulgidus* VC-16. The tree was constructed with the

maximum-likelihood method (Poisson correction model) and is bootstrapped with 50 replicates. Sequences of pyruvate formate lyase (Pfl) from *E. coli* were used as an outgroup (not shown).

Supplementary Figure 4 Phylogenetic relationship of identified genes in MAGs with currently-known molybdenum cofactor-containing hydrocarbon dehydrogenases. Reference sequences show the catalytic subunits of characterized cymene dehydrogenase (CmdA), alkane C_2-methylene hydroxylase (AhyA), and ethylbenzene dehydrogenase (EbdA/EbdA2) enzymes from other hydrocarbon-degrading bacteria. The tree was constructed with the maximum-likelihood method (Poisson correction model) and is bootstrapped with 50 replicates. Sequences of perchlorate reductase (PcrA) from *Dechloromonas aromatica* RCB were used as an outgroup (not shown).

Supplementary Figure 5 Protein presence/absence matrix for benzoyl-CoA anaerobic biodegradation pathway. The MAGs were shown only if it was at least partially complete (presence of at least three subunits within one cluster for BcrABCD). Presence of genes is indicated by blue boxes. Gene names: Bcr, benzoyl-CoA reductase; Oah, 6-oxo-cyclohex-1-ene-carbonyl-CoA hydrolase; Dch, cyclohex-1,5-diencarbonyl-CoA hydratase; Had, 6-hydroxycyclohex-1-ene-1-carbonyl-CoA dehydrogenases. More details about these functional genes and pathways can be found in the text and in Supplementary Data 4.

Supplementary Figure 6 Protein presence/absence matrix for reductive acetyl-CoA (Wood-Ljungdahl) pathway. Presence of genes is indicated by blue boxes.Columns correspond to the following enzymes: 1, formate dehydrogenase (Fhd) / formylmethanofuran dehydrogenase (Fwd); 2, formate-tetrahydrofolate synthetase (Fhs) / formylmethanofuran:tetrahydromethanopterin formyltransferase (Ftr); 3, methylene-

tetrahydrofolate dehydrogenase (FolD) / N5,N10-methenyltetrahydromethanopterin cyclohydrolase (Mch); 4, methylene-tetrahydrofolate dehydrogenase (FolD) / methylenetetrahydromethanopterin dehydrogenase (Mtd); 5, methylenetetrahydrofolate reductase (Met) / N5,N10-methylenetetrahydromethanopterin reductase (Mer), 6, acetyl-CoA synthetase (Acs), 7 carbon monoxide dehydrogenase (Cdh).

Supplementary References

Supplementary Note 1

Among all identified phyla, candidate phylum TA06 is the only one not yet given provisional names. Also known as GN04 or AC1, it was originally discovered in a hypersaline microbial mat [1]. First genomic representatives of this phylum were recovered from estuarine sediments [2] with a small number of other MAGs recently reported to belong to this lineage [3, 4]. Due to the paucity of available MAGs and misclassifications based on 16S rRNA gene sequences, members of TA06 are often 'confused' with members of the phylum WOR-3 (*Stahlbacteria*) [4]. In addition to the phylogenetic inference here based on 43 concatenated protein marker genes (Figure 2), the placement of two bins within the original TA06 phylum is further supported by genome classification based on concatenation of 120 ubiquitous, single-copy marker genes [5] as well as classification of 16S rRNA genes using the SILVA database [6] (Supplementary Data 2 and 3).

Supplementary Note 2

We performed a geochemical analysis of sediment porewater extracts. High concentrations of sulfate (Site E26: 16.55 mM; Site E29: 27.23 mM; Site E44: 25.63 mM) were detected at each of the three sites, consistent with sulfate being 28 mM in seawater and diffusing into sediments where it is consumed by sulfate reduction under anoxic conditions. H_2 and acetate concentrations were both below limits of detection (1 μM and 2.5 μM, respectively); this is consistent with previous observations in deep-sea sediments showing that H_2 and acetate are present at low steady-state concentrations due to tight coupling between producers and consumers [7, 8].

Supplementary Table 1 Sample metadata and shotgun sequencing results.

ID	*Site E26*	*Site E29*	*Site E44*
Latitude (N)	26.59	27.43	26.28
Longitude (W)	87.51	86.01	86.81
Geographic region	Henderson and Lund	Lloyd Ridge	Henderson and Lund
Water depth (km)	2.8	3.2	3.0
Quality-filtered reads	85 825 930	148 908 270	138 795 692
Contigs (> 500 bp)	168 069	700 804	695 891
Total size (bp)	149 863 977	738 738 047	755 473 006
Max contig (bp)	52 576	191 288	346 360
Average length (bp)	891	1 054	1 085
Curated MAGs	9	37	36

Supplementary Table 2 Alpha diversity estimates based on 16S rRNA amplicon sequencing.

	Richness Estimates			Diversity Indices			
Locations	Observed	Chao1	ACE	Shannon	Simpson	Inversed Simpson	Fisher
16S amplicons of bacterial communities							
Site E26	345	359 ± 7	356 ± 9	5.15	0.99	83.2	84.6
Site E29	1055	1374 ± 41	1483 ± 21	5.97	0.99	135.9	412.4
Site E44	348	360 ± 6	355 ± 9	5.28	0.99	100.8	85.6
16S amplicons of archaeal communities							
Site E26	192	195 ± 3	196 ± 7	4.22	0.96	23.3	43.7
Site E29	178	180 ± 2	181 ± 7	4.33	0.97	30.4	39.7
Site E44	220	247 ± 12	241 ± 7	4.21	0.96	25.6	52.2

Supplementary Table 3 Functional capacity of sediments based on normalized raw read count of genes (counts per million reads, CPM) encoding key metabolic enzymes.

ID	Site E26	Site E29	Site E44
AssA-Like	233	394	346
Canonical AssA	9	37	10
NmsA	0	0	0
BssA	2	4	2
AcsB	1485	2819	2474
DsrA	357	603	548
FeFe	332	380	335
NiFe Group 1	416	431	296
NiFe Group 2	55	5	23
NiFe Group 3	538	766	683
NiFe Group 4	73	99	92
Fe	0	0	0

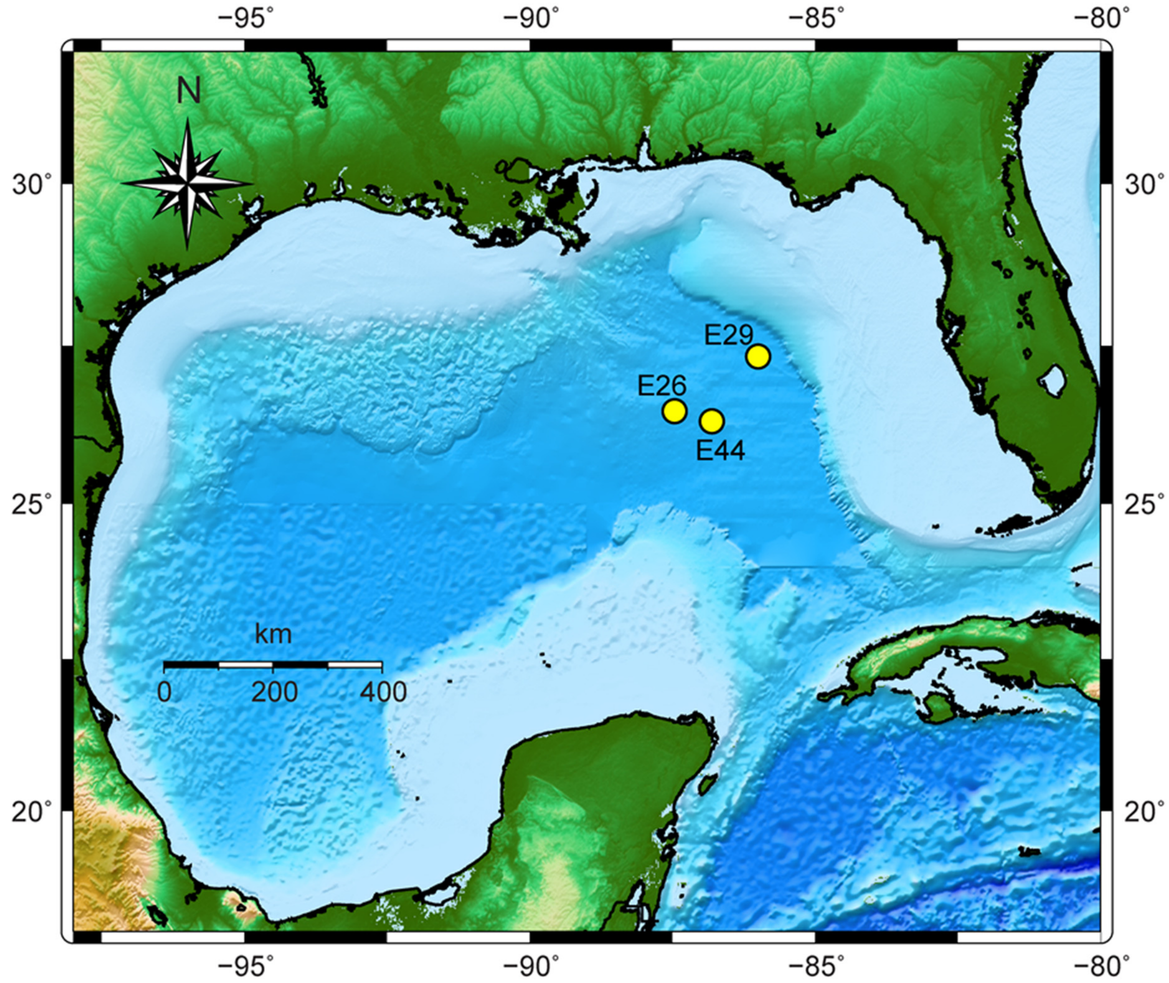

Supplementary Figure 1 Map of the Eastern Gulf of Mexico (GoM) showing the studied three sampling locations (E26, E29 and E44) and bathymetry of the study area.

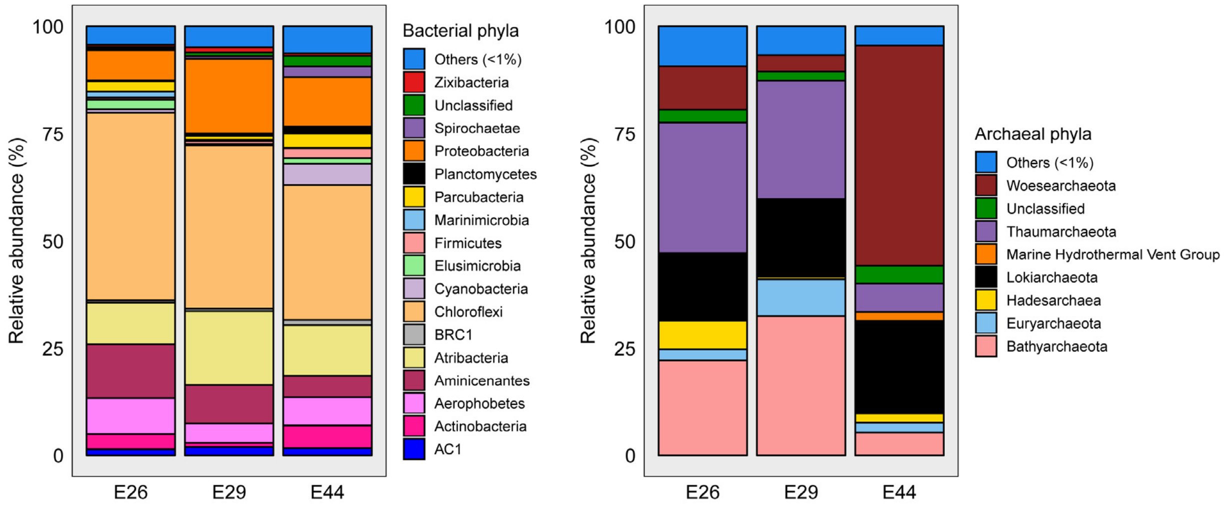

Supplementary Figure 2 Phylum-level community composition of classified bacterial and archaeal16S rRNA gene reads from separate bacterial and archaeal amplicon libraries. Left panel: bacterial composition; right panel: archaeal composition. Detailed numbers can be found in Supplementary Data 1.

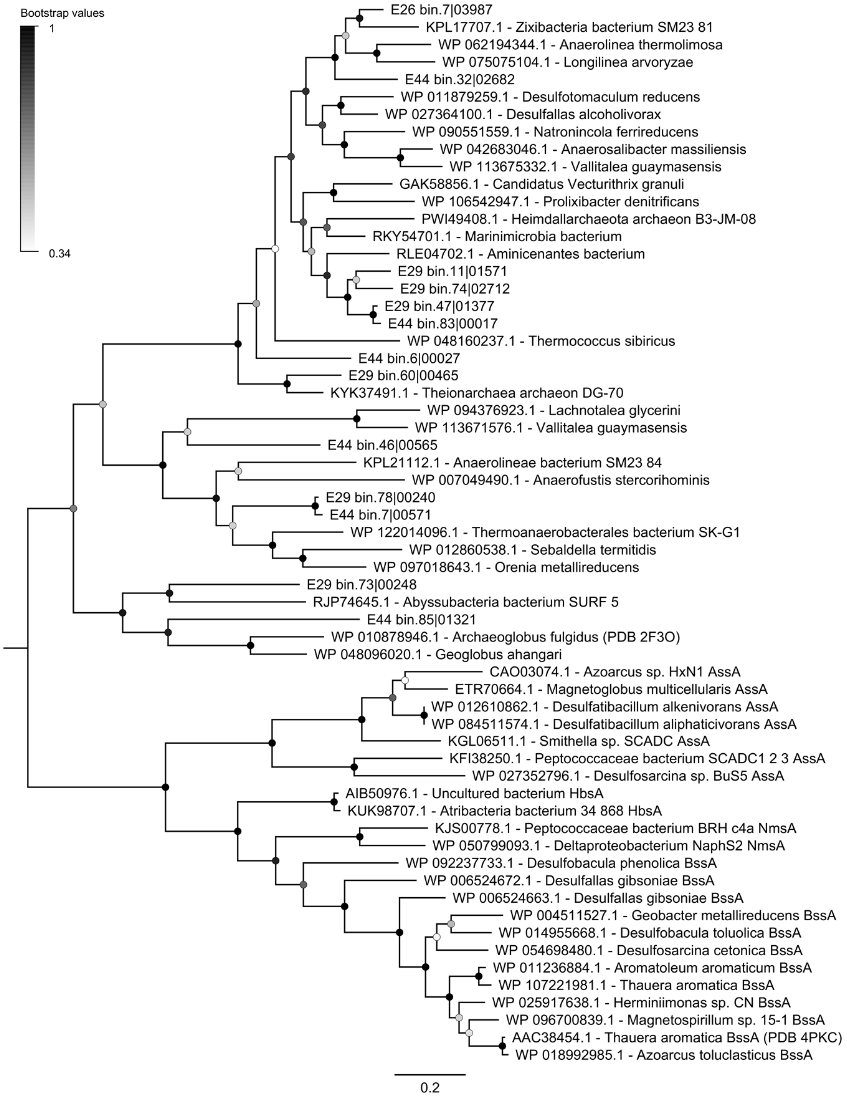

Supplementary Figure 3 Phylogenetic relationship of putative glycyl-radical enzymes in MAGs with those of akyl-/arylalkylsuccinate synthases. Reference sequences show canonical alkane succinate synthase (AssA), 4-hydroxylbenzyl succinate synthase (HbsA), benzyl succinate synthase (BssA), and homologous putative alkane-degrading enzymes from *Vallitalea guaymasensis* L81 and *Archaeoglobus fulgidus* VC-16. The tree was constructed with the maximum-likelihood method (Poisson correction model) and is bootstrapped with 50 replicates. Sequences of pyruvate formate lyase (Pfl) from *E. coli* were used as an outgroup (not shown).

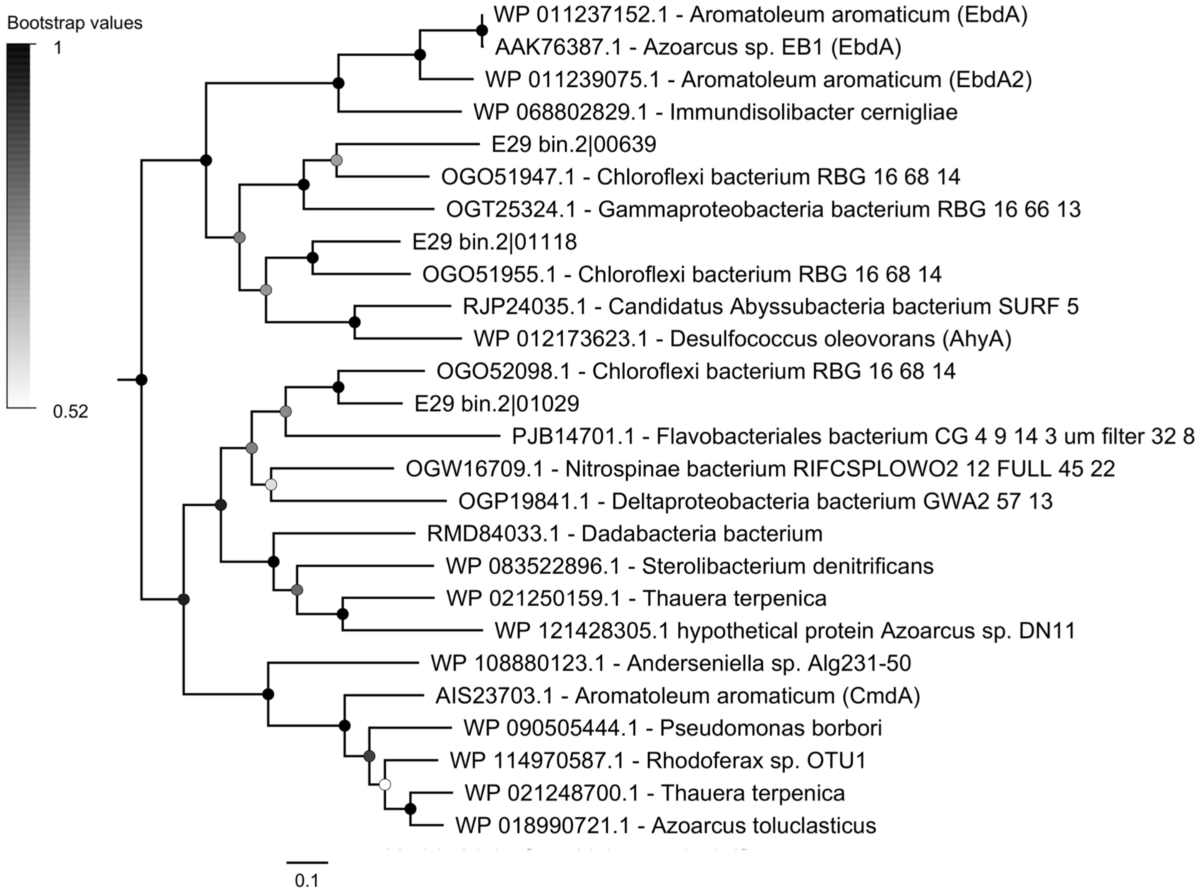

Supplementary Figure 4 Phylogenetic relationship of identified genes in MAGs with currently-known molybdenum cofactor-containing hydrocarbon dehydrogenases. Reference sequences show the catalytic subunits of characterized cymene dehydrogenase (CmdA), alkane C_2-methylene hydroxylase (AhyA), and ethylbenzene dehydrogenase (EbdA/EbdA2) enzymes from other hydrocarbon-degrading bacteria. The tree was constructed with the maximum-likelihood method (Poisson correction model) and is bootstrapped with 50 replicates. Sequences of perchlorate reductase (PcrA) from *Dechloromonas aromatica* RCB were used as an outgroup (not shown).

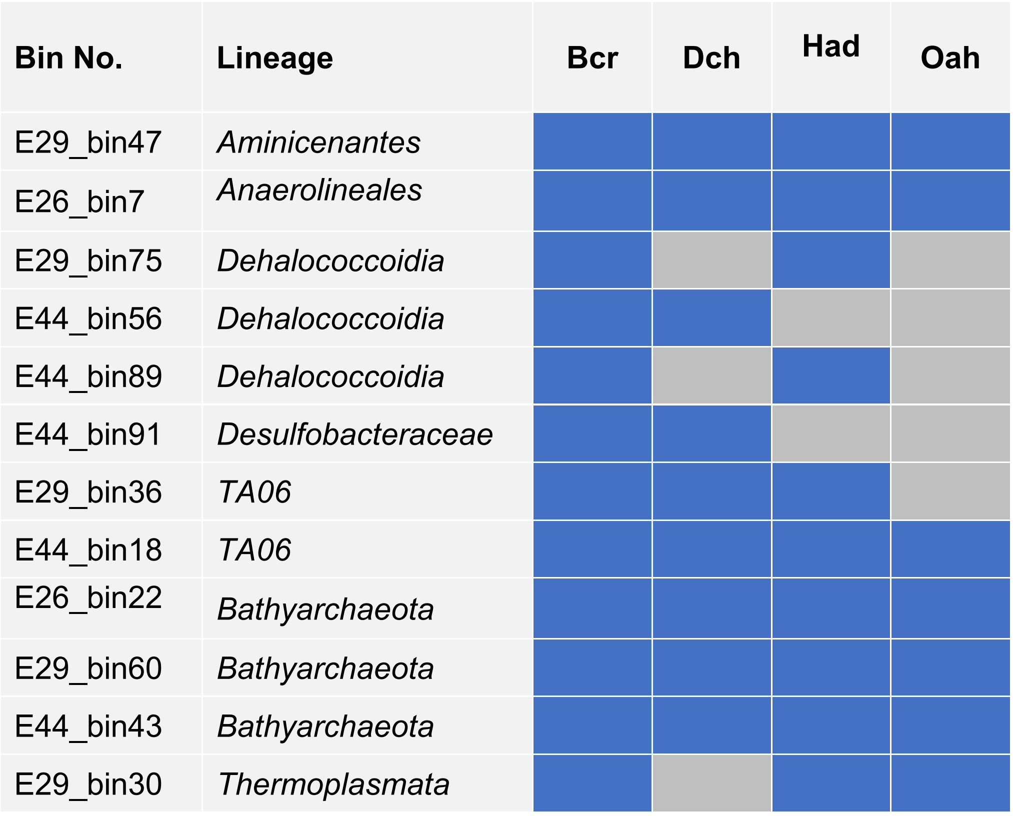

Supplementary Figure 5 Protein presence/absence matrix for benzoyl-CoA anaerobic biodegradation pathway. The MAGs were shown only if it was at least partially complete (presence of at least three subunits within one cluster for BcrABCD). Presence of genes isindicated by blue boxes. Gene names: Bcr, benzoyl-CoA reductase; Oah, 6-oxo-cyclohex-1-ene-carbonyl-CoA hydrolase; Dch, cyclohex-1,5-diencarbonyl-CoA hydratase; Had, 6-hydroxycyclohex-1-ene-1-carbonyl-CoA dehydrogenases. More details about these functional genes and pathways can be found in the text and in Supplementary Data 4.

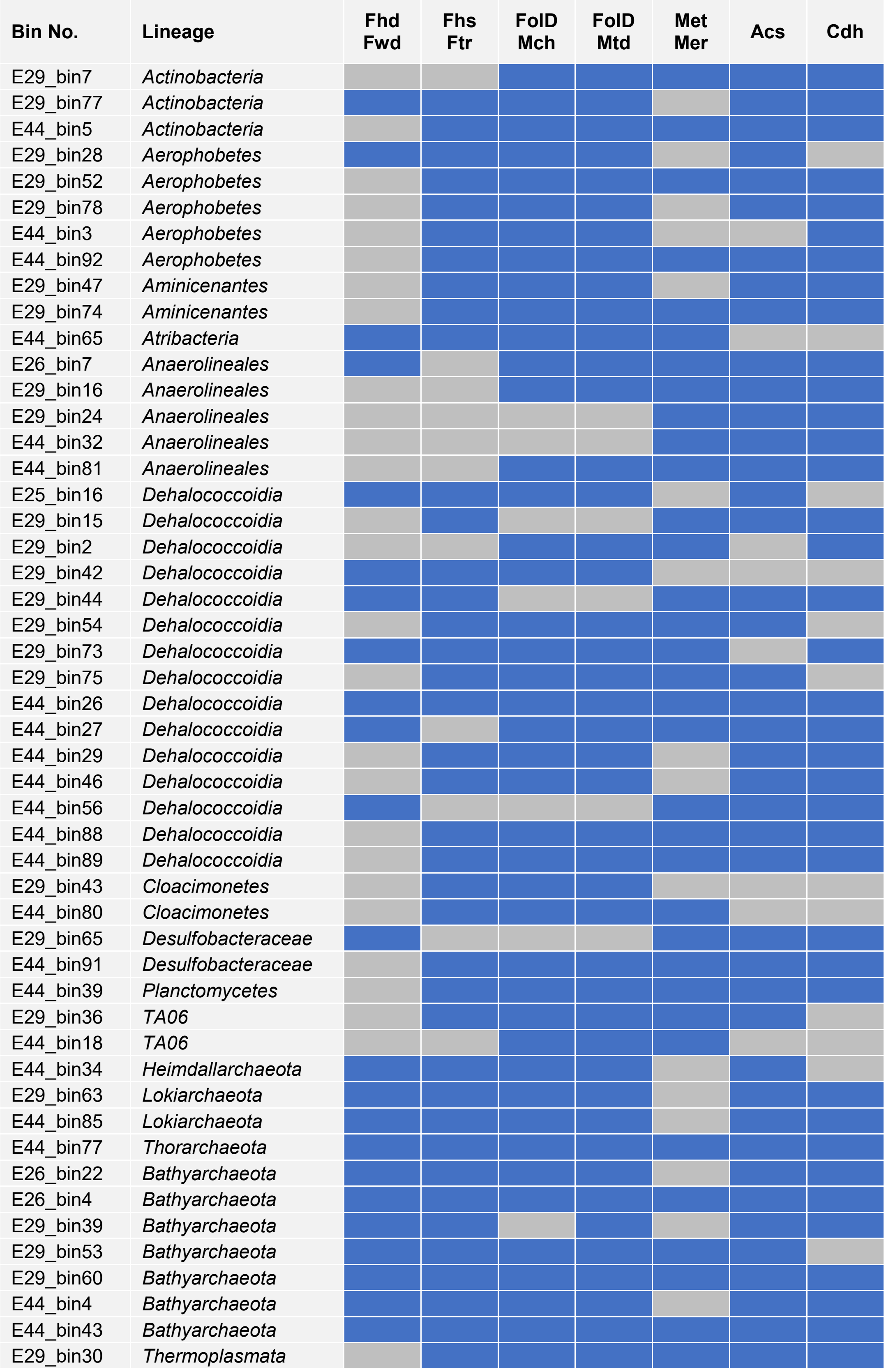
Bin No.
Lineage
Fhd Fwd
Fhs Ftr
FolD Mch
FolD Mtd
Met Mer
Acs
Cdh
E29_bin7 Actinobacteria
E29_bin77 Actinobacteria
E44_bin5 Actinobacteria
E29_bin28 Aerophobetes
E29_bin52 Aerophobetes
E29_bin78 Aerophobetes
E44_bin3 Aerophobetes
E44_bin92 Aerophobetes
E29_bin47 Aminicenantes
E29_bin74 Aminicenantes
E44_bin65 Atribacteria
E26_bin7 Anaerolineales
E29_bin16 Anaerolineales
E29_bin24 Anaerolineales
E44_bin32 Anaerolineales
E44_bin81 Anaerolineales
E25_bin16 Dehalococcoidia
E29_bin15 Dehalococcoidia
E29_bin2 Dehalococcoidia
E29_bin42 Dehalococcoidia
E29_bin44 Dehalococcoidia
E29_bin54 Dehalococcoidia
E29_bin73 Dehalococcoidia
E29_bin75 Dehalococcoidia
E44_bin26 Dehalococcoidia
E44_bin27 Dehalococcoidia
E44_bin29 Dehalococcoidia
E44_bin46 Dehalococcoidia
E44_bin56 Dehalococcoidia
E44_bin88 Dehalococcoidia
E44_bin89 Dehalococcoidia
E29_bin43 Cloacimonetes
E44_bin80 Cloacimonetes
E29_bin65 Desulfobacteraceae
E44_bin91 Desulfobacteraceae
E44_bin39 Planctomycetes
E29_bin36 TA06
E44_bin18 TA06
E44_bin34 Heimdallarchaeota
E29_bin63 Lokiarchaeota
E44_bin85 Lokiarchaeota
E44_bin77 Thorarchaeota
E26_bin22 Bathyarchaeota
E26_bin4 Bathyarchaeota
E29_bin39 Bathyarchaeota
E29_bin53 Bathyarchaeota
E29_bin60 Bathyarchaeota
E44_bin4 Bathyarchaeota
E44_bin43 Bathyarchaeota
E29_bin30 Thermoplasmata

Supplementary Figure 6 Protein presence/absence matrix for reductive acetyl-CoA (Wood-Ljungdahl) pathway. Presence of genes is indicated by blue boxes. Columns correspond to the following enzymes: 1, formate dehydrogenase (Fhd) / formylmethanofuran dehydrogenase (Fwd); 2, formate-tetrahydrofolate synthetase (Fhs) / formylmethanofuran:tetrahydromethanopterin formyltransferase (Ftr); 3, methylene-tetrahydrofolate dehydrogenase (FolD) / N5,N10-methenyltetrahydromethanopterin cyclohydrolase (Mch); 4, methylene-tetrahydrofolate dehydrogenase (FolD) / methylenetetrahydromethanopterin dehydrogenase (Mtd); 5, methylenetetrahydrofolate reductase (Met) / N5,N10-methylenetetrahydromethanopterin reductase (Mer), 6, acetyl-CoA synthetase (Acs), 7 carbon monoxide dehydrogenase (Cdh).

Supplementary references

1. Ley, R.E. et al. Unexpected diversity and complexity of the Guerrero Negro hypersaline microbial mat. *Appl Environ Microbiol* **72**, 3685-3695 (2006).
2. Baker, B.J., Lazar, C.S., Teske, A.P. & Dick, G.J. Genomic resolution of linkages in carbon, nitrogen, and sulfur cycling among widespread estuary sediment bacteria. *Microbiome* **3**, 14 (2015).
3. Hu, P. et al. Genome-resolved metagenomic analysis reveals roles for candidate phyla and other microbial community members in biogeochemical transformations in oil reservoirs. *MBio* **7**, e01669-01615 (2016).
4. Sieber, C.M.K. et al. Recovery of genomes from metagenomes via a dereplication, aggregation and scoring strategy. *Nat Microbiol* **3**, 836-843 (2018).
5. Parks, D.H. et al. A standardized bacterial taxonomy based on genome phylogeny substantially revises the tree of life. *Nat Biotechnol* (2018).
6. Quast, C. et al. The SILVA ribosomal RNA gene database project: improved data processing and web-based tools. *Nucleic Acids Res* **41**, D590-596 (2013).
7. Marshall, I.P.G., Karst, S.M., Nielsen, P.H. & Jørgensen, B.B. Metagenomes from deep Baltic Sea sediments reveal how past and present environmental conditions determine microbial community composition. *Mar Genomics* **37**, 58-68 (2018).
8. Ijiri, A. et al. Deep-biosphere methane production stimulated by geofluids in the Nankai accretionary complex. *Sci Adv* **4**, eaao4631 (2018).
