# Supplementary material for: Metabolic potential of uncultured bacteria and archaea associated with petroleum seepage in deep-sea sediments
Source: Nat Commun. 2019 Apr 18;10:1816. doi: 10.1038/s41467-019-09747-0 (PMC6472368; doi:10.1038/s41467-019-09747-0)
Supplement: Supplementary file 3 — Description of Additional Supplementary Files [file 41467_2019_9747_MOESM3_ESM.pdf]

Description of Additional Supplementary Files

File Name: Supplementary Data 1
Description: Relative abundance data for 16S rRNA gene amplicon sequencing and metagenome sequencing.

File Name: Supplementary Data 2
Description: Summary statistics for archaeal and bacterial MAGs.

File Name: Supplementary Data 3
Description: (1) GTDB-Tk classification of bins identified in this study as members of TA06; (2) Summary statistics of potential TA06 genomes based on NCBI and GTDB.

File Name: Supplementary Data 4
Description: Functional analysis of archaeal and bacterial MAGs. Presence/absence of genes are listed as: Presence: >1 (red), Absence: 0 (no color).

File Name: Supplementary Data 5
Description: Accession numbers for genes involved in anaerobic hydrocarbon degradation used as custom database.
