# Supplementary material for: Metabolic potential of uncultured bacteria and archaea associated with petroleum seepage in deep-sea sediments
Source: Nat Commun. 2019 Apr 18;10:1816. doi: 10.1038/s41467-019-09747-0 (PMC6472368; doi:10.1038/s41467-019-09747-0)
Supplement: Supplementary file 9 — Reporting Summary [file 41467_2019_9747_MOESM9_ESM.pdf]

nature research

Corresponding author(s): Xiyang Dong and Casey Hubert

Last updated by author(s): Mar 15, 2019

Reporting Summary

Nature Research wishes to improve the reproducibility of the work that we publish. This form provides structure for consistency and transparency in reporting. For further information on Nature Research policies, see Authors & Referees and the Editorial Policy Checklist.

Statistics

For all statistical analyses, confirm that the following items are present in the figure legend, table legend, main text, or Methods section.

n/a	Confirmed	
☐	☒	The exact sample size (*n*) for each experimental group/condition, given as a discrete number and unit of measurement
☐	☒	A statement on whether measurements were taken from distinct samples or whether the same sample was measured repeatedly
☒	☐	The statistical test(s) used AND whether they are one- or two-sided<br>*Only common tests should be described solely by name; describe more complex techniques in the Methods section.*
☒	☐	A description of all covariates tested
☒	☐	A description of any assumptions or corrections, such as tests of normality and adjustment for multiple comparisons
☒	☐	A full description of the statistical parameters including central tendency (e.g. means) or other basic estimates (e.g. regression coefficient) AND variation (e.g. standard deviation) or associated estimates of uncertainty (e.g. confidence intervals)
☒	☐	For null hypothesis testing, the test statistic (e.g. *F*, *t*, *r*) with confidence intervals, effect sizes, degrees of freedom and *P* value noted<br>*Give P values as exact values whenever suitable.*
☒	☐	For Bayesian analysis, information on the choice of priors and Markov chain Monte Carlo settings
☒	☐	For hierarchical and complex designs, identification of the appropriate level for tests and full reporting of outcomes
☒	☐	Estimates of effect sizes (e.g. Cohen's *d*, Pearson's *r*), indicating how they were calculated

Our web collection on statistics for biologists contains articles on many of the points above.

Software and code

Policy information about availability of computer code

Data collection

GC with Flame Ionization Detector
Dionex ICS-5000 reagent-free ion chromatography system
Perkin-Elmer Model LS 50B fluorometer
GC/MS
Finnigan MAT 252 isotope mass spectrometry
Thermo RS3000 HPLC fitted with an Ultimate 3000 UV detector
MAVEN
Ultra High-Performance Liquid Chromatography (UHPLC) using a hydrophilic interaction liquid chromatography column (Syncronis HILIC, Thermo Fisher)
MiSeq benchtop sequencer
NextSeq 500 System

Data analysis

metaSPAdes version 3.11.0
BBmap version 36
MetaBAT version 2.12.1
RefineM version 0.0.22
CheckM version 1.0.8
Prodigal
GhostKOALA tool
KEGG GENES database
Pfam, TIGRfam and custom HMM databases (https://github.com/banfieldlab/metabolic-hmms)
MetaErg (https://sourceforge.net/projects/metaerg/)
dbCAN web server
BLASTp
MEROPS database release 12.0

RAxML version 8
NCBI GenBank
MEGA7
FastTree version 2.1.9
hydrogenase classifier HydDB
FphyloFlash version 3.1 (https://hrgv.github.io/phyloFlash/)
SILVA SSU 132 rRNA database
GraftM
BLASTx

For manuscripts utilizing custom algorithms or software that are central to the research but not yet described in published literature, software must be made available to editors/reviewers. We strongly encourage code deposition in a community repository (e.g. GitHub). See the Nature Research guidelines for submitting code & software for further information.

Data

Policy information about availability of data

All manuscripts must include a data availability statement. This statement should provide the following information, where applicable:

- Accession codes, unique identifiers, or web links for publicly available datasets
- A list of figures that have associated raw data
- A description of any restrictions on data availability

DNA sequences (amplicon sequences, genomes and raw sequence reads) have been deposited in the NCBI BioProject database with accession number PRJNA415828 and PRJNA485648 (https://www.ncbi.nlm.nih.gov/bioproject/). The authors declare that all other data supporting the findings of this study are available within the article and its supplementary information files, or from the corresponding authors upon request.

Field-specific reporting

Please select the one below that is the best fit for your research. If you are not sure, read the appropriate sections before making your selection.

☐ Life sciences ☐ Behavioural & social sciences ☒ Ecological, evolutionary & environmental sciences

For a reference copy of the document with all sections, see nature.com/documents/nr-reporting-summary-flat.pdf

Ecological, evolutionary & environmental sciences study design

All studies must disclose on these points even when the disclosure is negative.

Study description	In this study, we used culture-independent approaches to study the role of microbial communities in the degradation of organic matter, including both detrital biomass and petroleum hydrocarbons. We performed metagenomic, geochemical and metabolomic analyses of deep seabed sediments (water depth ~3 km). Samples were chosen from three sites exhibiting evidence of different levels of migrated thermogenic hydrocarbons.
Research sample	The three marine sediment samples used in this study were chosen from among several sites sampled as part of a piston coring seafloor survey in the Eastern Gulf of Mexico. On the basis of TSF and UCM concentration thresholds, core segments from E26 and E29 were qualified and core segments from E44 was disqualified for unambiguous occurrence of thermogenic liquid hydrocarbons. Additionally, interstitial hydrocarbon gases were observed in the core segments of E29.
Sampling strategy	Piston cores typically penetrating 5 to 6 mbsf were sectioned in 20 cm intervals on board the research vessel immediately following piston core retrieval. Three intervals from the bottom half of the core were chosen for geochemical analysis, and were either frozen immediately (for liquid hydrocarbon analyses), or flushed with N2 and sealed in hydrocarbon-free gas tight metal canisters then frozen until analysis (for gaseous hydrocarbon analysis).
Data collection	X.D. and C.G. processed the data, reconstructed the genomes and performed the genome analyses. X.D., J.D. and D.M. performed thermodynamics analysis. M.C. confirmed phylogenetic analyses of genomes. A.C. and C.L. conducted amplicon sequencing and analyses. J.M.B. and B.B.B. collected samples and performed geochemistry analyses. J.E.R., R.A.G. and I.A.L. performed metabolomics analyses and data interpretation.
Timing and spatial scale	Marine surface sediments (0–20cm below seafloor) were collected from 174 locations in the Eastern Gulf of Mexico during January–March, 2011, aboard RV GeoExplorer as part of TDI Brooks International's Surface Geochemical Exploration (SGE) program.
Data exclusions	No data were excluded from the manuscript.
Reproducibility	For all the measured parameters by the instruments, they were all performed in at least for duplicates. For all other data prodcued by the software, they were obtained automatically.
Randomization	For samples for each site, they were homogenized and sub-sampled for the various treatments.
Blinding	As our study describes microbial processes and all data were collected using automated, digital instruments, blinding was not necessary in our experimental design.

Did the study involve field work? ☒ Yes ☐ No

Field work, collection and transport

Field conditions	The three marine sediment samples used in this study were chosen from among several sites sampled as part of a piston coring seafloor survey in the Eastern Gulf of Mexico.
Location	For samples E26: 26.59N, 87.51W, water depth of 2.8km<br>For samples E29: 27.43N, 86.01W, water depth of 3.2km<br>For samples E44: 26.28N, 86.81W, water depth of 3.0km
Access and import/export	Suitable footwear, gloves and equipments were used during the sampling process to minimize anthropogenic effects.
Disturbance	Disturbance was negligible as only very small volumes of samples were collected.

Reporting for specific materials, systems and methods

We require information from authors about some types of materials, experimental systems and methods used in many studies. Here, indicate whether each material, system or method listed is relevant to your study. If you are not sure if a list item applies to your research, read the appropriate section before selecting a response.

Materials & experimental systems

n/a	Involved in the study	
☒	☐	Antibodies
☒	☐	Eukaryotic cell lines
☒	☐	Palaeontology
☒	☐	Animals and other organisms
☒	☐	Human research participants
☒	☐	Clinical data

Methods

n/a	Involved in the study	
☒	☐	ChIP-seq
☒	☐	Flow cytometry
☒	☐	MRI-based neuroimaging
